# Monitoring Yellow Mealworm (*Tenebrio molitor*) as a Potential Novel Allergenic Food: Effect of Food Processing and Matrix

**DOI:** 10.3390/nu15030482

**Published:** 2023-01-17

**Authors:** Caterina Villa, Mónica B. M. V. Moura, Carla S. S. Teixeira, Joana Costa, Isabel Mafra

**Affiliations:** REQUIMTE-LAQV, Faculdade de Farmácia, Universidade do Porto, Rua de Jorge Viterbo Ferreira, 228, 4050-313 Porto, Portugal

**Keywords:** *Tenebrio molitor*, insects, novel allergens, real-time PCR, food matrix, heat processing

## Abstract

The consumption of insects has increased in western countries, raising concerns about their potential to induce food allergic reactions in sensitized/allergic individuals. This work intended to develop a real-time PCR approach for the detection/quantification of yellow mealworm (*Tenebrio molitor*) as a potential allergenic food in complex matrices. For this purpose, reference mixtures simulating the production of pork sausages and wheat biscuits containing known amounts of mealworm were used. Real-time PCR with TaqMan probe targeting the cytochrome b gene of *T. molitor* was able to detect up to 2 fg of insect DNA, and 1.0 and 0.1 mg/kg of mealworm flour in autoclaved sausages and baked biscuits, respectively. Generally, the method showed acceptable analytical performance parameters, confirming its suitability/applicability for a wide range of foods. However, real-time PCR data showed significant differences among food matrix and processing, highlighting the importance of using appropriate calibration models for quantitative analysis. Finally, the real-time PCR approach was successfully validated with blind mixtures and applied to commercial samples, demonstrating its efficacy and reliability in the quantification of mealworm in processed foodstuffs.

## 1. Introduction

Presently, the demand for alternative sources of animal-derived dietary proteins is increasing in parallel with the world’s growing population, requiring sustainable production of safe and nutritious food [1]. Edible insects are considered a valuable source of nutrients such as polyunsaturated fatty acids, essential amino acids, micronutrients, and proteins, having a nutritional value comparable or superior to those of both chicken and beef [2,3]. Additionally, they present a lower negative environmental impact than conventional animal-derived protein sources, with less greenhouse gas emissions and water pollution and higher feed conversion efficiency [4,5]. Therefore, in the near future, insects are expected to become important nutrient sources for animal and human consumption in western countries [5,6].

According to Regulation (EU) 2015/2283, edible insects are considered a novel food because they are “a food that was not used for human consumption to a significant degree within the European Union (EU) before 15 May 1997” [7]. Therefore, prior to the introduction of insect-derived products in the market and to guarantee their safety to consumers, the EU imposed a specific control for a complete risk assessment, with subsequent approvals taking place through a centralized procedure [8]. In the EU, several insect species are considered potential sources for food applications and human consumption, including *Acheta domesticus* (house-cricket), *Gryllus assimilis* (Jamaican field cricket), *Gryllodes sigillatus* (tropical house-cricket), *Locusta migratoria* (migratory locust), *Tenebrio molitor* (yellow mealworm), *Alphitobius diaperinus* (lesser mealworm), *Galleria mellonella* (greater wax moth), *Bombyx mori* (domestic silk moth), and *Hermetia illucens* (black soldier fly) [9,10]. Among them, recently, the assessment for human intake of yellow mealworm (*T. molitor*) did not raise any safety concerns by the EFSA Panel on Nutrition, Novel Foods and Food Allergens (NDA), being the first completed evaluation of an insect-derived food product [11]. However, the same panel considered that the consumption of the yellow mealworm might induce primary sensitization and allergic reactions to new IgE-binding proteins and/or cause allergic reactions in subjects with an allergy to crustaceans and dust mites (pan-allergens) [11].

Primary sensitization to edible insect allergens is relatively common via inhalation or skin contact, though their action as primary sensitizers through ingestion is still being evaluated. One major allergenic risk derives from the possible occurrence of cross-reactions with the arthropod pan-allergens, namely, tropomyosin and arginine kinase, which are present in crustaceans and mollusks [4,12]. Ribeiro et al. [13] listed several studies assessing the cross-reactivity between crustaceans and edible insects, all of them being attributed to tropomyosin or arginine kinase, the allergens causing clinically relevant symptoms. In *T. molitor*, tropomyosin, α-amylase, arginine kinase, and hexamerin were identified as the major offending food allergens [14]. Presently, these allergenic proteins are not part of the official WHO/IUIS list of allergens (http://www.allergen.org, accessed on 15 September 2022); although, yellow mealworm tropomyosin has been added to the ALLERGOME database (http://www.allergome.org, accessed on 15 September 2022) under the designation of Ten m 7.

These facts raised the need to develop strategies for the traceability of yellow mealworm in foods. Its detection as a potentially allergenic ingredient allows verifying labeling compliance and protecting the health of allergic/sensitized individuals. Currently, only few methodologies are available to detect yellow mealworm in foods, mostly relying on real-time PCR [15,16,17]. However, despite their demonstrated specificity for *T. molitor* or multiple insect detection, none of the methods provide quantitative analysis and application to food matrices. Recently, proteomic approaches using liquid chromatography with tandem mass spectrometry detection (LC–MS/MS) have characterized *T. molitor* allergens and provided preliminary insights on their identification but, again, without quantitative analysis and applicability to foods [18,19].

In the present work, a real-time PCR assay with a TaqMan probe, targeting the cytochrome b mitochondrial gene, was proposed as the first quantitative approach for yellow mealworm in processed foods. Raw and processed mixtures of sausages and biscuits containing *T. molitor* were used as model foods for the development of two quantitative models, which were assessed for their analytical performance. The influence of food matrix and processing on yellow mealworm detection was evaluated regarding eventual inhibitory effects and DNA degradation, respectively. The proposed quantitative models were validated with blind mixtures and further applied to commercial food samples to verify labeling compliance.

## 2. Materials and Methods

### 2.1. Sampling

Samples of yellow mealworm (*T. molitor*), house cricket (*A. domesticus*), migratory locust (*L. migratoria*), and black soldier fly (*H. illucens*) were acquired in Portuguese specialized companies. Larvae of *T. molitor* and *H. illucens* were obtained dehydrated, while adult specimens of *L. migratoria* and *A. domestica* were acquired boiled and dehydrated, respectively. Six commercial foods containing yellow mealworm (4 samples of protein bars and 2 chocolates) were acquired at local supermarkets. Insect and food samples were grounded and homogenized separately using a laboratory knife mill (Grindomix GM200, Retsch, Haan, Germany) with different containers and knives previously treated with DNA decontamination solution. Ground insect specimens and samples were immediately stored at −20 °C until DNA extraction.

Sixty-three different animal and plant species commonly used as food—namely, meats (boar, duck, partridge, hare, pheasant, deer, rabbit, chicken, turkey, lamb, ostrich, cow, horse, pork), seafood (horse mackerel, ling, mackerel, mussel, razor clam, squid, clam, cuttlefish, wedge clam, crab, brown crab, lobster, shrimp, crayfish, Argentine shrimp, octopus, snails), peanut and tree nuts (almond, hazelnut, cashew, pinion, chestnut), fruits (pineapple, bitter orange, nectarine, cherry tomato), and other plants (black mustard, chia, linseed, white sesame, pea, bean, chickpea, potato, rice, rapeseed, sunflower, corn, soybean, wheat, pumpkin, oat, barley, rye, lupine, cassava, wheat pasta, fava bean)—were used for specificity testing.

### 2.2. Preparation of Reference and Validation Model Mixtures

For method development and to assess the effects of food matrix and processing, two independent sets of reference model mixtures containing known amounts of *T. molitor* flour (100,000 mg/kg; 10,000 mg/kg; 1000 mg/kg; 100 mg/kg; 10 mg/kg; 1 mg/kg; and 0.1 mg/kg (*w*/*w*)) were prepared to simulate the industrial production of (i) pork sausages and (ii) wheat biscuits. For pork sausages, an initial mixture containing 500 g of minced pork meat, 300 g of lard, 200 g of crushed ice, and 16 g of salt was prepared. The biscuit dough was made by mixing 400 g of butter, 400 g of sugar, 8 eggs, 1 kg of wheat flour, and 480 g of water.

The first spiked levels containing 100,000 mg/kg (10%) of yellow mealworm were prepared by the addition of 15.0 g of insect powder to 135.0 g of pork sausage mixture or by the addition of 20.0 g of insect powder to 180.0 g of biscuit dough. The following mixtures were further prepared by successive additions of the respective food matrix (sausage or biscuit) until reaching the level of 0.1 mg/kg (0.00001%). Similarly, two sets of blind samples were prepared for method validation with the following concentrations: 40,000 mg/kg; 20,000 mg/kg; 4000 mg/kg; 2000 mg/kg; 400 mg/kg; and 40 mg/kg (*w/w*) of insect powder in pork sausages and 40,000 mg/kg; 4000 mg/kg; 400 mg/kg; and 40 mg/kg (*w/w*)) of insect in wheat biscuits.

Reference model and validation mixtures were divided into 2 portions: (i) one portion of each spiking level, including blank matrices (0% of yellow mealworm), was immediately frozen and stored at −20 °C, as raw matrices; (ii) the second portion was heat-treated by autoclaving at 121 °C for 15 min to simulate the production of canned sausages or by oven-baking at 180 °C for 20 min to simulate biscuits. The heat-treated reference mixtures were grounded, homogenized, and stored as previously described for insect material and food samples.

### 2.3. DNA Extraction

DNA extraction of model mixtures and animal material, including *T. molitor*, *A. domestica*, and *H. illucens*, was performed with the NucleoSpin^®^ Food kit (Macherey-Nagel, Düren, Germany) according to the manufacturer’s instructions with minor alterations. The NucleoSpin^®^ Plant II kit (Protocol 2) (Macherey-Nagel, Düren, Germany) was used to extract DNA from plant species, following the manufacturer’s instructions with minor adjustments. Due to some difficulties in extracting amplifiable DNA from crustaceans and *L. migratoria*, the SureFood Prep Advanced kit (Protocol 1) (R-Biopharm AG, Darmstadt, Germany) was used for these cases according to the manufacturer’s instructions with some modifications. For all kits, the minor adjustments included the use of 200 mg of each sample (starting material) with the addition of 2 µL of RNase (2 mg/mL) after the cell lysis step. All the extractions were performed at least in duplicate, and the extracts were stored at −20 °C until further analysis.

Yield and purity of extracts were evaluated by UV spectrophotometry on a Synergy HT multimode microplate reader (BioTek Instruments, Inc., Winooski, VT, USA), using a Take 3 plate accessory of reduced volume (micro-volume scale). The DNA content was assessed by the nucleic acid quantification protocol with the sample type defined for double-strand DNA in the Gen5 data analysis software version 2.02 (BioTek Instruments, Inc., Winooski, VT, USA).

### 2.4. Selection of Yellow Mealworm Genetic Markers

For the specific detection of *T. molitor*, several sequences from mitochondrial and nuclear genes were selected from NCBI database and evaluated in silico. The complete mitochondrial genome of *T. molitor* (accession no. KP994554.1) was analyzed, from which four regions were selected as candidates for primer design, namely, NADH dehydrogenase subunit 2 (NADH2), cytochrome C oxidase subunit 1 (COX1), NADH dehydrogenase subunit 5 (NADH5), and cytochrome b (cytb) (Table 1). Two nuclear regions, encoding tropomyosin mRNA (accession no. MK425158.1) and E Cadherin mRNA (accession no. DQ988044), were also selected for primer design (Table 1). The basic local alignment search tool Blast (https://blast.ncbi.nlm.nih.gov/Blast.cgi accessed on 15 September 2022) was applied to each selected region to identify potential marker sequences with no or very low local similarity with homologue sequences of different species. After selecting the best marker for yellow mealworm identification, a hydrolysis probe targeting the cytb gene was designed for real-time PCR amplification of *T. molitor* (Table 1).

Primer specificity was confirmed using the Primer-BLAST tool (http://www.ncbi.nlm.nih.gov/tools/primer-blast/ accessed on 15 September 2022) while primer and probe properties (absence of hairpins and self-hybridization) were checked by Oligo Calc software (http://biotools.nubic.northwestern.edu/OligoCalc.html accessed on 15 September 2022).

The primers 18SEU-F/18SEU-R, designed in the conserved region of the 18S rRNA gene, were used to amplify a 140 bp fragment as an endogenous control of eukaryotes (Table 1) [20].

The oligonucleotide primers and hydrolysis probe were synthesized by Eurofins Genomics (Ebersberg, Germany).

### 2.5. Qualitative PCR

Qualitative PCR amplifications were performed in a total reaction volume of 25 µL, containing 2 µL of DNA extract (20 ng), ultrapure water (Sigma-Aldrich, Steinheim, Germany), 1 × buffer (670 mM of Tris-HCl (pH 8.8), 16 mM of (NH_4_)_2_SO_4_, 0.1% of Tween 20), 200 µM of each dNTP (Grisp, Porto, Portugal), 1.0 U of SuperHot Taq DNA Polymerase (Genaxxon Bioscience, Ulm, Germany), 2.5 mM of MgCl_2_, and 200 nM of each primer (Table 1). The PCR runs were carried out in an MJ Mini thermal cycler (BioRad Laboratories, Hercules, CA, USA) or in a SimpliAmp™ Thermal Cycler (Applied Biosystem™, ThermoFisher Scientific, Waltham, MA, USA) with the following temperature programs: initial denaturation at 95 °C for 5 min; 40 cycles at 95 °C for 30 s (except for YMW_CytB-F/YMW_CytB-R with 36 cycles), 58 °C (for YMW_ND2-F/R, YMW_ND5-F/R, and YMW_COX1-F/R), 59 °C (for YMW_TM-F/R and YMW_CytB-F/R), or 61 °C (for YMW_CAD-F/R) (Table 1) for 30 s and 72 °C for 30 s; and a final extension at 72 °C for 5 min.

The amplification capacity of DNA extracts was confirmed by qualitative PCR using the same components as above but with 1.5 mM of MgCl_2_ and 400 nM of universal eukaryotic primers 18SEU-F/18SEU-R (Table 1), following the described temperature program: initial denaturation at 95 °C for 5 min; 35 cycles at 95 °C for 30 s, 60 °C for 30 s, and 72 °C for 30 s; and a final extension at 72 °C for 5 min.

All PCR amplicons were verified by electrophoresis in a 1.5% agarose gel stained with 1 × GelRed (Biotium, Inc., Hayward, CA, USA) and carried out in 1 × SGTB (Grisp, Porto, Portugal) for 25–30 min at 200 V. The agarose gel was visualized under a UV light tray GelDoc™ EZ System (Bio-Rad Laboratories, Hercules, CA, USA), and a digital image was recorded using Image Lab software version 5.2.1 (Bio-Rad Laboratories, Hercules, CA, USA).

### 2.6. Real-Time PCR

Real-time PCR assays were performed using reaction mixtures of 20 μL, containing 1 × SsoFast Probes Supermix (Bio-Rad Laboratories, Hercules, CA, USA), 240 nM of each primer (YMW_CytB-F/R), 160 nM of probe (YMW_CytB-P), and 2 µL of DNA extract (20 ng). The amplifications were carried out in a fluorometric thermal cycler CFX96 Real-time PCR Detection System (Bio-Rad Laboratories, Hercules, CA, USA) with the following conditions: 95 °C for 5 min; 45 cycles at 95 °C for 10 s, 59 °C for 10 s, and 72 °C for 30 s with the collection of fluorescence signal at the end of each cycle. The software Bio-Rad CFX Manager 3.1 (Bio-Rad Laboratories, Hercules, CA, USA) was used to acquire and process the data obtained from each real-time PCR run. Cycles of quantification (Cq), also known as cycle threshold (Ct) values, were calculated using the software threshold settings defined at 150. Real-time PCR trials were repeated in two independent runs using *n* = 4 replicates in each one.

### 2.7. Statistical Analysis

Statistical analysis was performed with GraphPad Prism version 8.0.2 software (GraphPad Software, San Diego, CA, USA) to evaluate the significance of differences between the Cq values of distinct matrices and processing treatments (raw/autoclaved sausages and raw/baked biscuits) at the same spiked level, using a significance level of 0.05 (95% of confidence interval). Data with normal distribution, as determined by Shapiro–Wilk test, were further processed with one sample *t*-test and ordinary one-way ANOVA (Tukey’s multiple comparisons test). Data following a non-normal distribution were analyzed by a non-parametric Mann–Whitney test and Kruskal–Wallis test (Dunn’s multiple comparisons test). Significant differences were considered when *p* < 0.05, which are represented by * or different letters to express *t*-test or ANOVA results, respectively.

## 3. Results

### 3.1. Specificity and Sensitivity Evaluation

Six sequences from mitochondrial and nuclear genes of yellow mealworm, namely, NADH2, COX1, NADH5, cytb, tropomyosin mRNA, and E Cadherin mRNA, were in silico evaluated favorably as candidate markers for its detection. Six primer sets were then designed and experimentally tested for their sensitivity and specificity (Table 1). To evaluate the absolute sensitivity by qualitative PCR, the DNA extracts of *T. molitor* were 10-fold serially diluted with quantities ranging from 20 ng to 0.2 pg. All assays produced PCR fragments with the expected size, presenting absolute sensitivities between 2 pg and 0.2 pg of *T. molitor* DNA, from which the COX1 (117 bp), NADH5 (180 bp), and cytb (160 bp) targets achieved the highest sensitivity levels (Figure S1, Supplementary material). For method development, the specificity of the proposed primers for *T. molitor* identification was a key issue to assess, which was initially determined by testing the potential reactivity with the most common edible insects, namely, *H. illucens*, *L. migratoria*, and *A. domestica* (Table S1, Supplementary material). The PCR results with primers targeting the NADH2 and COX1 regions showed high reactivity with *H. illucens*, while NADH5 primers reacted with *A. domesticus*, evidencing a lack of specificity for yellow mealworm identification. The remaining primers targeting the tropomyosin, cytb, and E Cadherin genes did not react with any of the non-target insect species (Table S1, Supplementary material). Among them, the YMW_CytB-F/R primers were selected as the most promising for method development because they provide the highest sensitivity (0.2 pg of DNA) and specificity, without any cross-reactivity with the tested animal and plant species commonly used as food (Table S1, Supplementary material).

For the estimation of the relative sensitivity of PCR with YMW_CytB-F/R primers, reference model mixtures of mealworm in sausages and biscuits were tested, producing PCR products down to 1 mg/kg (0.0001%) and 10 mg/kg (0.001%) of the insect in raw and processed model mixtures, respectively (Figure S2, Supplementary material).

### 3.2. Development of Real-Time PCR Systems

**Figure 1:**
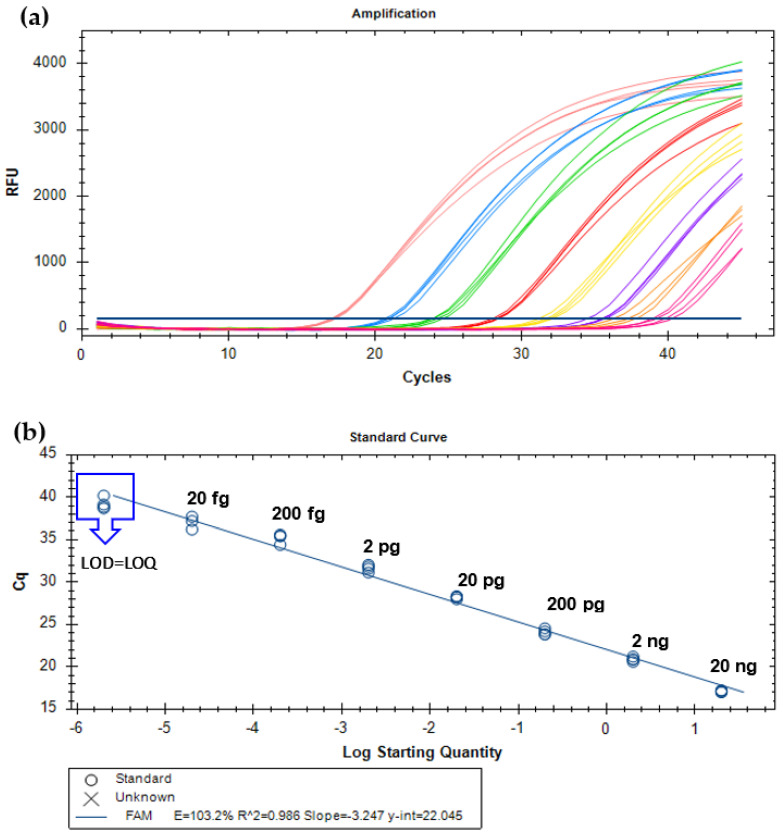
Amplification curves (**a**) and respective calibration curve (**b**) obtained by real-time PCR with TaqMan probe targeting the cytochrome b gene and using serially diluted (1/10) DNA extracts of *T. molitor* from 20 ng to 2 fg (*n* = 4 replicates). Cq—cycle of quantification.

After demonstrating the specificity and high sensitivity of the new primers (YMW_CytB-F/R) targeting the cytb gene of *T. molitor*, a new specific TaqMan probe was designed for the development of a real-time PCR assay. As for qualitative PCR, the absolute sensitivity was determined with the amplification of 10-fold serially diluted yellow mealworm DNA (from 20 ng to 2 fg, 8 levels) by real-time PCR. Each concentration level was amplified in quadruplicate, at least in two independent PCR runs. Figure 1 presents the amplification curves (Figure 1A) and respective calibration curve (Figure 1B) of one example run. The assay enabled the amplification of all replicates (*n* = 8) until the level of 2 fg of *T. molitor* DNA, which was established as the limit of detection (LOD), defined as the lowest concentration at which 95% of the replicates are detected [21,22]. The limit of quantification (LOQ) was considered equal to the LOD since it was within the linear range of the calibration curve, which covered 7 orders of magnitude, above the ideal of 5 or 6 log_10_ concentrations [21,22]. The established criteria for assessing the performance of real-time PCR assays, namely, the PCR efficiency (90 and 110%); the slope, within −3.6 and −3.1; and the correlation coefficient (*R*^2^), above 0.98, were also considered [21,22]. The obtained average parameters of two PCR runs (PCR efficiency of 102.3 ± 1.4%, slope of 3.269 ± 0.031, *R*^2^ of 0.984 ± 0.004) underline the high analytical performance of the assay because they were all within the acceptance criteria.

**Figure 2:**
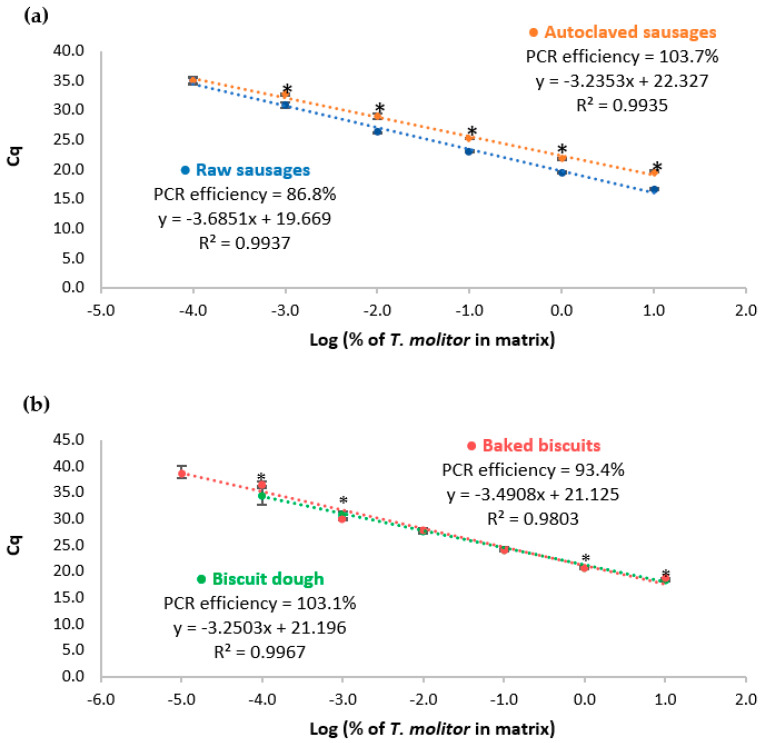
Calibration curves obtained by real-time PCR with TaqMan probe targeting cytochrome b gene using sausages (**a**) and biscuits (**b**) model mixtures before and after thermal treatment, containing 100,000 mg/kg; 10,000 mg/kg; 1000 mg/kg; 100 mg/kg; 10 mg/kg; 1 mg/kg; and 0.1 mg/kg (*w*/*w*) of mealworm (*n* = 8 replicates). For the same spiking level, * means statistically significant differences between raw and processed mixtures (*p* < 0.05), following *t*-test analysis.

The proposed real-time PCR system targeting the cytb gene was then assayed with reference mixtures of pork sausages and wheat biscuits containing known amounts of yellow mealworm, before and after thermal treatment, to provide a practical calibration model. Figure 2 shows the obtained calibration curves and the respective performance data are summarized in Table 2. According to the abovementioned criteria, the developed system generally presents acceptable performance parameters, namely, slope ranging from −3.6851 to −3.2353, *R*^2^ of 0.9809–0.9967, and PCR efficiency within 86.8–103.7%. It can be noted that raw sausages presented a PCR efficiency (86.8%) slightly below the acceptance criteria (90%), which can be due to the high enzymatic activity, increasing the instability of the raw matrix compared with the processed one, thus decreasing the overall DNA integrity. A sensitivity of 1 mg/kg (0.0001%) of yellow mealworm in sausages (raw and autoclaved) and biscuits before thermal treatment was achieved. However, the highest sensitivity was obtained with baked biscuits as model mixtures, reaching an LOD of 0.1 mg/kg (0.00001%) of yellow mealworm. In all cases, values for LOQ were established as equal to the LOD because they were within the linear range of the respective calibration curve [21,22].

According to the literature, reliable and highly sensitive methods for the quantitative detection of yellow mealworm in processed foodstuffs are still scarce. Debode, Marien, Gérard, Francis, Fumière, and Berben [15] proposed a real-time PCR method for the qualitative detection of *T. molitor* based on the wingless and the cadherin genes, reaching an LOD of 10.4 pg of insect DNA. A set of multiplex PCR assays for the detection of nine edible insects was developed by Tramuta, Gallina, Bellio, Bianchi, Chiesa, Rubiola, Romano, and Decastelli [17] targeting the 16S rRNA gene, which, in the case of *T. molitor* detection, achieved a sensitivity of up to 12.5 pg of DNA. However, both methods applied conventional PCR and serial dilutions of genomic DNA without any quantification of the target gene or species. The use of model mixtures to simulate the addition of the target species to a food matrix is imperative for a realistic and practical determination of its content. In fact, all the components of the food matrix, such as fats, carbohydrates, and other metabolites can affect the DNA extraction, leading to a decrement in PCR efficiency and affecting the quantitative results [23]. More recently, Köppel, Schum, Habermacher, Sester, Piller, Meissner, and Pietsch [16] established a real-time PCR assay with SYBR Green dye for the simultaneous detection of three edible insects, including yellow mealworm. The system targeting the cytochrome oxidase I region of the tested insects was able to detect up to 0.1% (1000 mg/kg) of mealworm in biscuits and hamburgers model mixtures. Comparing the herein developed real-time PCR approach with the reported studies, the highest sensitivity for mealworm detection was achieved in the present work, down to 2 fg of *T. molitor* DNA and 1.0 and 0.1 mg/kg of mealworm flour in autoclaved sausages and baked biscuits, respectively.

It has already been reported that the consumption of insects or insect-based products can induce the development of allergic reactions in patients previously sensitized to crustaceans and/or to insects via inhalation/skin [24,25]. However, consistent data about the prevalence, severity, exposure assessment, and potency of insect allergens are still limited, making the establishment of a minimum eliciting dose a challenging task [26]. Recently, Garino, Mielke, Knüppel, Selhorst, Broll, and Braeuning [27] identified a possible eliciting dose (ED05) for the ingestion of *T. molitor*, suggesting that 121 mg of the insect (0.402 g in the case of mealworm burgers or 1.205 g of insect energy bars) could be able to elicit a clinical response in shrimp-sensitized individuals. The method developed by Köppel, Schum, Habermacher, Sester, Piller, Meissner, and Pietsch [16] can detect 100 mg of insect in a serving size portion of 100 g (1000 mg/kg) of food, which is very close to the eliciting dose suggested by Garino et al. [27]. The herein developed method provides a much superior sensitivity in both tested matrices because it detects 0.01–0.1 mg of insect in a serving size portion of 100 g of food, thus efficiently protecting at least 95% of the crustacean-allergic population.

### 3.3. Validation of the Method

To validate the quantitative performance of the proposed real-time PCR method, two sets of blind mixtures containing between 40,000 and 40 mg/kg of yellow mealworm were analyzed; the results are summarized in Table 3. The performance parameters of trueness, expressed as bias and considered acceptable within ± 25.0% of the actual value over the tested dynamic range, and precision, expressed as the coefficient of variation (CV) with acceptable values ≤ 25%, were estimated for each level of concentration [21,22]. Regarding the system using model mixtures of sausages as calibrants, most of the obtained values of CV were between 3.2% and 15.3%, except for the lowest concentration of 40 mg/kg of mealworm in sausages without thermal treatment (#F) (Table 3). The obtained bias values for sausage blind mixtures were mostly within the acceptable criteria (–24.9 to 22.7%), except for the lowest tested level of 40 mg/kg #F (229.8%) and #L (–30.5%). In the case of the biscuit model mixtures, bias values were acceptable for all raw and most baked mixtures, excepting for the concentration of 4000 mg/kg (#R). Precision values were all acceptable for biscuit mixtures, excluding the mixture #P (32.3%). At low estimation levels, it is expected to obtain a general lower reliability, which explains the results for mixtures #F, #L, and #P, all at the lowest concentration of 40 mg/kg (0.004%) of yellow mealworm in matrix (Table 3). However, the high bias value of mixture #R (40.6%) can be due to some effect of thermal treatment (baking) on the quantification of yellow mealworm at this concentration. In general, both food matrices used as calibration models were suitable for mealworm quantification since most of the obtained precision and trueness data were within the acceptable criteria.

### 3.4. Thermal Processing and Matrix Effects

**Figure 3:**
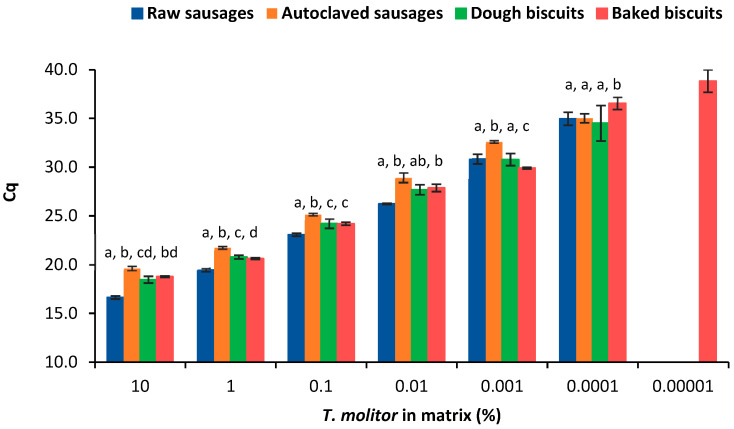
Comparison of the dynamic range of the calibration curves obtained by real-time PCR targeting cytochrome b gene according to the type of thermal treatment and food matrix (mean ± SD of *n* = 8 replicates from 2 independent runs). Different letters for the same spiking level mean statistically significant differences between matrices (*p* < 0.05) following ordinary one-way ANOVA or ANOVA analysis.

The effects of thermal treatment and food matrix on the detection of yellow mealworm were assessed by comparing the calibration curves obtained with both sets of reference mixtures in sausages and biscuits before and after thermal treatment (Figure 2 and Figure 3, Table 2). Data show that autoclaving did not affect the sensitivity as the LOD was the same value (1 mg/kg) for raw and autoclaved sausages. For baked biscuits, the LOD was lower (0.1 mg/kg) compared with that of raw dough. These facts can be explained by a decrease in the enzymatic activity and water content (positive effect on homogeneity) induced by oven-baking; stabilizing the food matrix; and, consequently, facilitating DNA extraction, as already reported [28,29,30].

As previously highlighted (Section 3.2), the efficiency of the real-time PCR assay seemed to improve after the application of autoclaving to sausage matrix. Comparing real-time PCR data of raw and processed reference sausages, the obtained Cq values were significantly different (*p* < 0.05) in all spiking levels, excluding the last level of 1 mg/kg of insect (Figure 2A). These results suggest that the harsh processing conditions of autoclaving (combination of high temperature and pression) contributed to the degradation of the target DNA to some extent, leading to differences of 2–3 amplification cycles for the same spiking level. When compared with the other model mixtures, the autoclaved sausages presented a delayed amplification of the target DNA (Figure 2 and Figure 3) but maintained the sensitivity of the method—a fact that has already been reported in other works [31]. On the other hand, the effect of oven-baking in biscuit dough was not so evident because the differences in the Cq values were, in general, below 0.5, being considered as significantly different (*p* < 0.05) for the spiking levels of 100,000 mg/kg, 10,000 mg/kg, 10 mg/kg, and 1 mg/kg (Figure 2B).

To assess the effect of food matrix, the Cq values obtained in both food matrices, before and after processing, were also statistically compared regarding *T. molitor* detection (Figure 3). Generally, Cq values were significantly different between food matrices in both raw and processed model mixtures. As also demonstrated in Section 3.2, the highest sensitivity of the method was achieved in baked biscuits (Table 2, Figure 2), probably due to the elimination of water and subsequent reduction of enzymatic activity, thus stabilizing the matrix. Raw sausages amplified earlier than biscuit dough. This could be due to the higher high enzymatic activity of biscuit dough; although, the raw sausage mixtures possess more lipidic compounds that also affect PCR efficiency together with high enzymatic activity. The higher polysaccharide content of biscuit dough does not seem to affect amplification since the baked biscuits, which are more concentrated in these compounds, exhibited the best analytical performance. This fact clearly suggests that the food matrix composition can differently affect the target detection by real-time PCR, highlighting the importance of using appropriate calibration models that should simulate as close as possible the samples under analysis.

### 3.5. Analysis of Commercial Samples

The applicability of the developed method was tested with commercial food samples that only accounted for protein/chocolate bars containing yellow mealworm due to the scarcity of insect-containing foods in the Portuguese market. Prior to real-time PCR analysis, all the samples were assayed for their amplification capacity with universal eukaryotic primers (18SEU-F/R), followed by species-specific qualitative PCR that confirmed the presence of yellow mealworm (data not shown). The calibration model using baked biscuits was considered the most appropriate for quantitative analysis according to the composition/processing of the products under evaluation. Table 4 summarizes the relevant label information of the tested commercial samples and the quantitative real-time PCR results, including Cq values and the estimated insect content. *T. molitor* was detected in all the samples with estimated contents ranging from 1769 mg/kg (0.177%) to 23,925 mg/kg (2.39%), confirming the labeled information. Regarding the two chocolate samples that declared 0.2% of dehydrated *T. molitor* larvae, the estimated contents were both in full agreement with the labeled information as they were 0.227% and 0.243% for samples #5 and #6, respectively. Thus, these results prove the efficacy and robustness of the method for the quantification of mealworm in processed samples.

## 4. Conclusions

Generally, the advantages of using *T. molitor* as food are related with the supply of alternative protein-rich food sources; decreasing the livestock production; and, consequently, reducing the emissions of greenhouse gases. Additionally, its proteins are also sources of bioactive peptides with anti-hypertensive, anti-inflammatory, antioxidant, anti-diabetic, and anti-SARS-CoV−2 properties [32]. However, the consumption of insects such as *T. molitor* as a food source represents a health risk to sensitized/allergic individuals because its ingestion might induce allergic reactions to crustaceans and dust mites or due to primary sensitization to insect proteins. The health protection of allergic individuals demands the control of processed foods, which should rely on the development of highly specific and sensitive analytical methods for the detection of insects in foods [8].

Herein, the first quantitative real-time PCR method was proposed for the detection of *T. molitor* as a potential allergenic food in complex and processed matrices. After a careful evaluation of several candidate markers for the identification of yellow mealworm, the cytochrome b gene was selected as the most appropriate for method development in terms of sensitivity and specificity. The method allowed detecting *T. molitor* DNA down to 2 fg and, based on the use of reference mixtures, the approach provided calibration models with relative LOD/LOQ of 0.1–1.0 mg/kg, as affected by matrix and processing. The calibration models complied with the acceptance criteria for real-time PCR assays regarding dynamic range, slope, coefficient of correlation, and PCR efficiency. The calibration model using sausage mixtures reached an LOD/LOQ of 1.0 mg/kg, covering 5 orders of magnitude within the dynamic range of 10,000–1.0 mg/kg (10–0.0001%) of yellow mealworm in sausage matrix, which was significantly affected by autoclaving. The calibration model using biscuit mixtures provided the highest sensitivity (0.1 mg/kg) of yellow mealworm in dough and baked biscuits, presenting statistical differences between Cq values of raw dough and biscuits for most concentration levels. This finding proves that autoclaving can affect the performance of the method caused by DNA degradation during processing, while oven-baking stabilizes the matrix by reducing the enzymatic activity. The comparison of data of the two distinct mealworm-containing reference mixtures showed that the food matrix can also differently affect the target detection by real-time PCR. Raw biscuit dough showed slightly higher Cq values than raw sausage matrix, probably due to the relevant enzymatic activity of biscuit dough, despite the higher lipid content of raw sausage mixtures. For these reasons, the selection of appropriate calibration models that mimic the test samples as close as possible is a crucial task.

In general, the validation results using blind mixtures of both food matrices showed that the calibration models were suitable for quantitative analysis since most precision and trueness data were within the acceptance criteria. The real-time PCR method was then used to analyze commercial food samples of protein/chocolate bars with the calibration model of baked biscuits, which demonstrated the successful applicability of the approach, with estimated values according to the labeled statements. This result indicates that the analyzed samples of chocolates and proteins bars provided reliable information on the mealworm content, constituting a good reference for consumers potentially allergic to crustaceans. Therefore, a new highly sensitive and specific real-time PCR method is proposed to quantify *T. molitor* in processed foods, consisting of a valuable tool that can contribute to the effective allergen management and health protection of the sensitized/allergic consumers.

## Figures and Tables

**Table 1:** Key data of primers and the hydrolysis probe designed for yellow mealworm detection and universal eukaryotic primers targeting a conserved 18S rRNA.

Target Region	Primers	Sequence (5′→3′)	Amplicon (bp)	Annealing Temperature (°C)	Accession No. (NCBI)/ Reference
Nuclear 18S rRNA	18SEU-F 18SEU-R	TCTGCCCTATCAACTTTCGATGG TAATTTGCGCGCCTGCTG	140	60	[20]
NADH dehydrogenase subunit 2	YMW_ND2-F YMW_ND2-R	ACGTCTTTTCTACAGAAGCATC GGGGTTAATGAATTCGTTTGTTA	113	56	KP994554.1/
Cytochrome C oxidase subunit 1	YMW_COX1-F YMW_COX1-R	CCCATGGAGGAGCATCTGTC TGCCCTGTGGTCGTATGTTGA	117	60	KP994554.1
NADH dehydrogenase subunit 5	YMW_ND5-F YMW_ND5-R	AATTGACCAGCCTCCCCATAAA CTACACTTAGTCAGCTTGGTCT	180	56	KP994554.1
Tropomyosin mRNA	YMW_TM-F YMW_TM-R	ATGCTCAAGACAAGGCCGATGC TCCTTGTTGGCTTGTTCCAGGT	160	59	MK425158.1/
Cytochrome b	YMW_CytB-F YMW_CytB-R YMW_CytB-P	CCTTACCTAGGAACAACAATTGT GATTGTTTGATCCTGTTTGGTGT FAM-TTGAGGAGGATTTGCTGTAGACAATGCA-BHQ1	160	59	KP994554.1
E Cadherin mRNA	YMW_CAD-F YMW_CAD-R	TGGTTGATCGGTGTTTCGGTA AATCCCGACTCTTGCGACC	137	61	DQ988044

**Table 2:** Calibration curve parameters obtained by quantitative real-time PCR targeting cytochrome b gene, using model mixtures of sausages and biscuits containing *T. molitor*, before and after thermal treatment.

Parameter	Sausages		Biscuits	
	Raw	Autoclaved	Raw	Baked
Correlation coefficient (*R*^2^)	0.9937	0.9935	0.9967	0.9803
Slope	−3.6851	−3.2353	−3.2503	−3.4908
PCR efficiency (%)	86.8	103.7	103.1	93.4
Relative LOD/LOQ (mg/kg)	1.0	1.0	1.0	0.1

**Table 3:** Validation results of the real-time PCR system targeting the cytochrome b gene applied to blind mixtures of yellow mealworm in sausages and biscuits before and after thermal treatment.

Blind Mixtures	Actual *T. molitor* Content (mg/kg)	Estimated *T. molitor* Content (Mean ± SD, mg/kg) ^a^	CV ^b^ (%)	Bias ^c^ (%)
Sausages				
Raw				
A	40,000	47,249 ± 6034	15.1	18.1
B	20,000	25,912 ± 2009	10.0	−22.8
C	4000	4914 ± 291.9	7.3	22.9
D	2000	2230 ± 216.4	10.8	11.5
E	400	490.8 ± 61.1	15.3	22.7
F	40	131.9 ± 33.3	83.2	229.8
Autoclaved				
G	40,000	46,907 ± 2666	6.7	17.3
H	20,000	21,445 ± 1238	6.2	7.2
I	4000	3003 ± 126.8	3.2	−24.9
J	2000	2175 ± 237.3	11.9	8.8
K	400	330.4 ± 50.3	12.6	−17.4
L	40	27.80 ± 4.04	10.1	−30.5
Biscuits				
Raw				
M	40,000	39,620 ± 3674	9.2	−1.0
*n*	4000	3146 ± 324.7	8.1	−21.4
O	400	356.7 ± 29.2	7.3	−10.8
*p*	40	31.62 ± 12.91	32.3	−21.0
Oven-baked				
Q	40,000	45,322 ± 2359	5.2	13.3
R	4000	5623 ± 276.7	4.9	40.6
S	400	311.0 ± 23.3	7.5	−22.3
T	40	33.68 ± 6.06	18.0	−15.8

^a^ Mean values ± standard deviation (SD) of replicate assays (*n* = 8) of two independent runs; ^b^ CV, coefficient of variation; ^c^ Bias = (mean value − true value)/true value × 100.

**Table 4:** Results of the application of the quantitative real-time PCR using biscuits model mixtures to detect and quantify yellow mealworm in commercial samples.

Samples	Relevant Label Information	YMW_CytB-F/R (Cq ± SD) ^a^	Estimated *T. molitor* Content (Mean ± SD) ^b^	
			mg/kg	%
Protein bars				
1	Contains dried yellow flour larvae (*T. molitor* larvae)	22.89 ± 0.08	2482 ± 160	0.248 ± 0.016
2	Contains dried yellow flour larvae (*T. molitor* larvae)	21.76 ± 0.11	5231 ± 197	0.523 ± 0.020
3	Contains dried yellow flour larvae (*T. molitor* larvae)	23.40 ± 0.12	1769 ± 104	0.177 ± 0.010
4	Contains dried yellow flour larvae (*T. molitor* larvae)	19.45 ± 0.07	23,925 ± 1449	2.39 ± 0.14
Chocolates				
5	Contains dehydrated *T. molitor* larvae (0.2%)	26.54 ± 0.85	2270 ± 110	0.227 ± 0.011
6	Contains dehydrated *T. molitor* larvae (0.2%)	22.82 ± 0.44	2426 ± 510	0.243 ± 0.051

^a^ Mean values of cycle of quantification (Cq) ± standard deviation (SD) of two independent runs (*n* = 8); ^b^ mean values ± SD, expressed in mg of *T. molitor* flour per kg of food and *w*/*w* percentage.

## Data Availability

Not applicable.

## Supplementary Materials

The following supporting information can be downloaded at: https://www.mdpi.com/article/10.3390/nu15030482/s1. Table S1: Resumed results of amplifiability with universal primers and reactivity testing of the designed primers for the intended target of mealworm with the most common edible insects and 63 plant/animal species; Figure S1: Agarose gel electrophoresis of PCR products using primers to specifically amplify yellow mealworm DNA; Figure S2: Agarose gel electrophoresis of PCR products targeting the cytochrome b gene (primers YMW_CytB-F/R) using DNA extracts of binary mixtures of yellow mealworm in sausages.
