# Supplementary material for: Monitoring Yellow Mealworm (Tenebrio molitor) as a Potential Novel Allergenic Food: Effect of Food Processing and Matrix
Source: Nutrients. 2023 Jan 17;15(3):482. doi: 10.3390/nu15030482 (PMC9921270; doi:10.3390/nu15030482)
Supplement: Supplementary file 1 [file nutrients-15-00482-s001.zip › nutrients-2132229-supplementary.pdf]

Table S1. Resumed results of amplifiability with universal primers and reactivity testing of the designed primers for the intended target of mealworm with the most common edible insects and 63 plant/animal species.

Species/Primers	18SEU-F/R	YMW_ND2-F/R	YMW_COX1-F/R	YMW_ND5-F/R	YMW_TM-F/R	YMW_CAD-F/R	YMW_CytB-F/R
Tenebrio molitor	+++	+++	+++	+++	+++	+++	+++
Insect species							
Acheta domesticus	+++	-	-	+	-	-	-
Locusta migratoria	+++	-	-	-	-	-	-
Hermetia illucens	+++	+++	+++	-	-	-	-
Plant species							
Black mustard	+++	NT	NT	NT	NT	NT	-
Chia	+++	NT	NT	NT	NT	NT	-
Linseed	+++	NT	NT	NT	NT	NT	-
White sesame	+++	NT	NT	NT	NT	NT	-
Pea	+++	NT	NT	NT	NT	NT	-
Bean	+++	NT	NT	NT	NT	NT	-
Chickpea	+++	NT	NT	NT	NT	NT	-
Potato	+++	NT	NT	NT	NT	NT	-
Rice	+++	NT	NT	NT	NT	NT	-
Rapeseed	+++	NT	NT	NT	NT	NT	-
Sunflower	+++	NT	NT	NT	NT	NT	-
Corn	+++	NT	NT	NT	NT	NT	-
Soybean	+++	NT	NT	NT	NT	NT	-
Wheat	+++	NT	NT	NT	NT	NT	-
Pumpkin	+++	NT	NT	NT	NT	NT	-
Oat	+++	NT	NT	NT	NT	NT	-
Barley	+++	NT	NT	NT	NT	NT	-
Rye	+++	NT	NT	NT	NT	NT	-
Lupine	+++	NT	NT	NT	NT	NT	-
Cassava	+++	NT	NT	NT	NT	NT	-
Wheat pasta	+++	NT	NT	NT	NT	NT	-
Fava beans	+++	NT	NT	NT	NT	NT	-
Pineapple	+++	NT	NT	NT	NT	NT	-
Bitter orange	+++	NT	NT	NT	NT	NT	-
Nectarine	+++	NT	NT	NT	NT	NT	-
Cherry tomato	+++	NT	NT	NT	NT	NT	-
Peanut	+++	NT	NT	NT	NT	NT	-
Almond	+++	NT	NT	NT	NT	NT	-
Hazelnut	+++	NT	NT	NT	NT	NT	-

Cashew	+++	NT	NT	NT	NT	NT	-
Pinion	+++	NT	NT	NT	NT	NT	-
Chestnut	+++	NT	NT	NT	NT	NT	-
Animal species							
Boar	+++	NT	NT	NT	NT	NT	-
Duck	+++	NT	NT	NT	NT	NT	-
Partridge	+++	NT	NT	NT	NT	NT	-
Hare	+++	NT	NT	NT	NT	NT	-
Pheasant	+++	NT	NT	NT	NT	NT	-
Deer	+++	NT	NT	NT	NT	NT	-
Wild rabbit	+++	NT	NT	NT	NT	NT	-
Chicken	+++	NT	NT	NT	NT	NT	-
Turkey	+++	NT	NT	NT	NT	NT	-
Lamb	+++	NT	NT	NT	NT	NT	-
Ostrich	+++	NT	NT	NT	NT	NT	-
Cow	+++	NT	NT	NT	NT	NT	-
Horse	+++	NT	NT	NT	NT	NT	-
Pig	+++	NT	NT	NT	NT	NT	-
Horse mackerel	+++	NT	NT	NT	NT	NT	-
Ling	+++	NT	NT	NT	NT	NT	-
Mackerel	+++	NT	NT	NT	NT	NT	-
Mussel	+++	NT	NT	NT	NT	NT	-
Razor clam	+++	NT	NT	NT	NT	NT	-
Squid	+++	NT	NT	NT	NT	NT	-
Clam	+++	NT	NT	NT	NT	NT	-
Cuttlefish	+++	NT	NT	NT	NT	NT	-
Wedge clam	+++	NT	NT	NT	NT	NT	-
Crab	+++	NT	NT	NT	NT	NT	-
Brown crab	+++	NT	NT	NT	NT	NT	-
Lobster	+++	NT	NT	NT	NT	NT	-
Shrimp	+++	NT	NT	NT	NT	NT	-
Crayfish	+++	NT	NT	NT	NT	NT	-
Argentine shrimp	+++	NT	NT	NT	NT	NT	-
Octopus	+++	NT	NT	NT	NT	NT	-
Snails	+++	NT	NT	NT	NT	NT	-

(+++) strong amplification band; (+) band less intense than the positive control; (+/-) faint band; (-) negative amplification. NT – not tested.

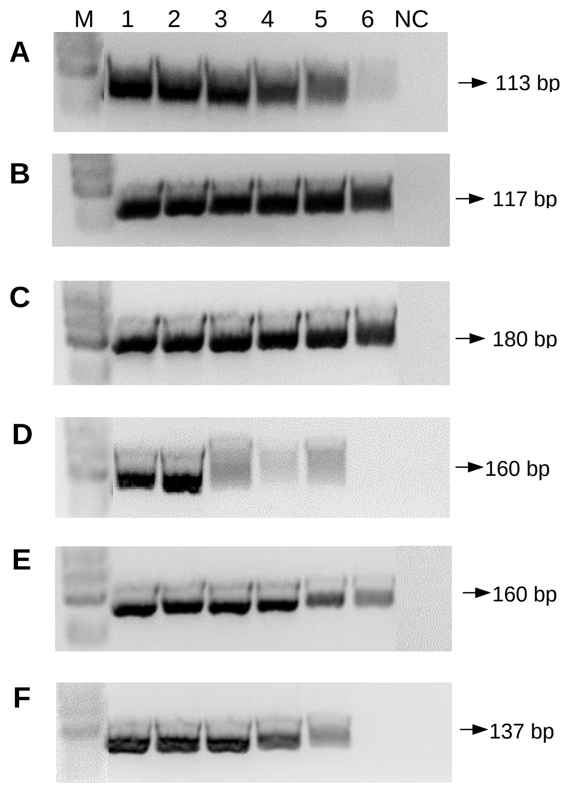

Fig. S1. Agarose gel electrophoresis of PCR products using primers to specifically amplify yellow mealworm DNA. (A) YMW_ND2-F/R targeting the NADH dehydrogenase subunit 2 gene, (B) YMW_COX1-F/R for the cytochrome C oxidase subunit 1 gene, (C) YMW_ND5-F/R for the NADH dehydrogenase subunit 5 gene, (D) YMW_TM-F/R for the tropomyosin gene, (E) YMW_CytB-F/R for the cytochrome b gene and (F) YMW_CAD-F/R for the E Cadherin gene. Legend: lane M, 100 bp DNA molecular marker (Bioron, Ludwigshafen, Germany); lane 1-6, 20 ng, 2 ng, 0.2 ng, 0.02 ng, 2 pg, 0.2 pg, respectively; NC, negative control.

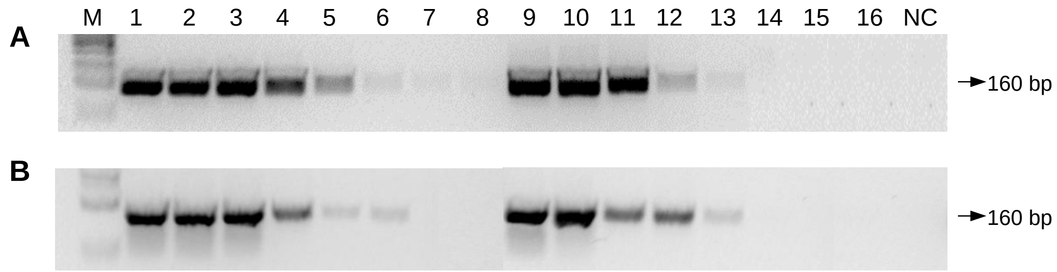

Fig. S2. Agarose gel electrophoresis of PCR products targeting the cytochrome b gene (primers YMW_CytB-F/R) using DNA extracts of binary mixtures of yellow mealworm in sausages (A) and biscuits (B), before (lanes 1-8) and after processing (lanes 9-16). Legend: lane M, 100 bp DNA molecular marker (Bioron, Ludwigshafen, Germany); lanes 1-7, 9-15, model mixtures containing 100,000 mg/kg (10%); 10,000 mg/kg (1%); 1,000 mg/kg (0.1%); 100 mg/kg (0.01%); 10 mg/kg (0.001%); 1 mg/kg (0.0001%) and 0.1 mg/kg (0.00001%) of mealworm in matrix, respectively; lanes 8 and 16, model mixtures without the addition of mealworm; NC, negative control.
